# Growth and repair factors, osteoactivin, matrix metalloproteinase and heat shock protein 72, increase with resolution of inflammation in musculotendinous tissues in a rat model of repetitive grasping

**DOI:** 10.1186/s12891-016-0892-3

**Published:** 2016-01-18

**Authors:** Nagat Frara, Samir M. Abdelmagid, Michael Tytell, Mamta Amin, Steven N. Popoff, Fayez F. Safadi, Mary F. Barbe

**Affiliations:** Department of Anatomy and Cell Biology, Temple University School of Medicine, Philadelphia, PA USA; Department of Stem Cell Biology and Regenerative Medicine, Lerner Research Institute, Cleveland Clinic, Cleveland, OH USA; Department of Neurobiology and Anatomy, Wake Forest University School of Medicine, Winston-Salem, NC USA; Department of Anatomy and Neurobiology, Northeast Ohio Medical University (NEOMED), Rootstown, OH USA

**Keywords:** Overuse, Osteoactivin, Metalloproteinases, Heat shock protein, Restorative repair, Muscle, Tendon

## Abstract

**Background:**

Expression of the growth factor osteoactivin (OA) increases during tissue degeneration and regeneration, fracture repair and after denervation-induced disuse atrophy, concomitant with increased matrix metalloproteinases (MMPs). However, OA’s expression with repetitive overuse injuries is unknown. The aim of this study was to evaluate: 1) OA expression in an operant rat model of repetitive overuse; 2) expression of MMPs; 3) inflammatory cytokines indicative of injury or inflammation; and 4) the inducible form of heat shock protein 70 (HSPA1A/HSP72) as the latter is known to increase during metabolic stress and to be involved in cellular repair. Young adult female rats performed a high repetition negligible force (HRNF) food retrieval task for up to 6 weeks and were compared to control rats.

**Methods:**

Flexor digitorum muscles and tendons were collected from 22 young adult female rats performing a HRNF reaching task for 3 to 6 weeks, and 12 food restricted control (FRC) rats. OA mRNA levels were assessed by quantitative polymerase chain reaction (qPCR). OA, MMP-1, -2, -3, and -13 and HSP72 protein expression was assayed using Western blotting. Immunohistochemistry and image analysis was used to evaluate OA and HSP72 expression. ELISA was performed for HSP72 and inflammatory cytokines.

**Results:**

Flexor digitorum muscles and tendons from 6-week HRNF rats showed increased OA mRNA and protein expression compared to FRC rats. MMP-1, -2 and -3 progressively increased in muscles whereas MMP-1 and -3 increased in tendons with HRNF task performance. HSP72 increased in 6-week HRNF muscles and tendons, compared to controls, and co-localized with OA in the myofiber sarcolemma. IL-1alpha and beta increased transiently in tendons or muscles in HRNF week 3 before resolving in week 6.

**Conclusion:**

The simultaneous increases of OA with factors involved in tissue repair (MMPs and HSP72) supports a role of OA in tissue regeneration after repetitive overuse.

## Background

Overuse injuries are now considered a leading cause of long-term pain and physical disability world-wide [1], with diagnoses including tendinopathies and muscle disorders [2, 3]. Studies have identified repetition and duration as two of the key risk factors for upper extremity overuse injuries [4]. Moreover, overuse injuries commonly occur as a result of prolonged repetitive loading of the muscle-tendon unit, even at low force levels [5, 6].

Several studies have shown that repetitive movements lead to tissue injury ([7] and as reviewed in [8]). Barbe and Barr have developed an operant rat model of upper extremity overuse in which rats learn to perform repetitive tasks, such as a high repetition negligible force (HRNF) food retrieval task. In this particular task, rats reach at a rate of 4 reaches/min into a portal to retrieve a 45-mg pellet of food for 2 h/day (in four 30 min sessions/day) for 3 days/week. Performance of this HRNF task leads to modest signs of myositis and tendinitis in forearm muscles and tendons and increased focal sites of myotendon fray and fibroblast proliferation [9, 10]. After such tissue damage, it is known that inflammatory cells infiltrate tissues, which, along with injured cells, produce inflammatory cytokines and other mediators that either exacerbate damage or assist in tissue repair [11, 12].

Osteoactivin (OA) is a type I transmembrane protein that is also known as glycoprotein nonmelanoma protein B (GPNMB). OA is a growth factor involved in tissue turnover during regeneration [13–15] and is known to up-regulate the expression of matrix metalloproteinases (MMPs) in fibroblasts infiltrating denervated muscle, leading to increased extracellular matrix (ECM) turnover [16]. OA increases in tissue matrices during fracture repair [13] and influences adhesion and migration of select cell types (including fibroblasts) that are involved in tissue repair [17], and regulates muscle regeneration in desmin-deficient cardiomyocytes [18]. In a process called ectodomain shedding, the extracellular fragments of OA are cleaved on the plasma membrane and released to the ECM where they act as cytokines or growth factors [19–21] in addition to increasing MMP production [14]. While it is clear that OA increases under several types of repair and regeneration conditions, its expression in association with overuse injuries has yet to be examined.

The inducible form of HSP70 (HSPA1A; commonly known as HSP72) mediates tissue repair after injury and protects skeletal muscle from atrophy, damage and dystrophy [22–26]. HSP70/72 plays a role in skeletal muscle repair or regeneration and adaptation after high-force eccentric exercise [27–29] and increases concomitant with MMP-2 in skeletal muscle following high intensity training [30], apparently acting together to promote muscle matrix remodeling [31]. Increased expression of HSP70 following muscle injury regulates both the early inflammatory and regenerative phases of muscle regeneration [32].

Our goals here were to evaluate the temporal and spatial expression of OA, MMPs and HSP72 in forelimb flexor digitorum muscles and tendons in rats performing a HRNF task for 3 to 6 weeks. Inflammatory cytokines were also examined in order to determine if injury/inflammatory processes were present in the tissues at the same time points. Although OA has yet to be studied in a model of overuse, we hypothesized that it would increase in the overloaded skeletal muscles and tendons, as would other known mediators of repair (MMPs and HSP70).

## Methods

### Animals

All experiments were approved by the Temple University Institutional Animal Care and Use Committee in compliance with NIH guidelines for the humane care and use of laboratory animals. Rats were housed individually in the central animal facility in transparent plastic cages in a 12 h light: 12 h dark cycle with free access to water. Studies were conducted on a total of 34 young adult (3.5 months of age at onset of experiments) Sprague-Dawley, female rats. All rats were handled for 1 week, and then food restricted to 80–90 % of their naïve full body weights for another week before onset of the experiment to motivate interest in 45 mg food pellets used for reward and retrieval (Banana flavored 45 mg food pellets; Bio-Serve, Flemington, NJ). All rats were weighed once to twice per week, provided regular rat chow daily and allowed to gain weight over the course of the experiments since they were young adult rats. All rats were maintained within 5–10 % of the weights of age-matched normal control rats (used only for weight matching purposes and therefore not included in the study). Rats were then randomly divided into food restricted control rats (*n* = 12) and high repetition negligible force (HRNF) task rats (*n* = 22) performing this task for 3 weeks (*n* = 12) and 6 weeks (*n* = 10).

### Behavioral task paradigm

The HRNF task has been described in detail previously [9]. Briefly, rats were trained for 5 min/day, 5 days/week for 10–12 days to learn to retrieve a 45-mg pellet of food from a shoulder-height portal at a reach rate of 4 reaches/min, making this a high repetition task (i.e. faster than 30 s/cycle) [33]. After this initial training period, task rats then performed the HRNF task for 2 h/day in four 30 min sessions/day on 3 days/week for 3 to 6 weeks in customized operant behavioral apparati (Med Associates, St Albans, VT), described in detail previously [9]. Retrieval of the 45 mg food pellet from the portal was estimated as <5 % maximum pulling force, making this a negligible force task [34]. Rats were allowed to use their preferred reach limb to reach and retrieve the food pellet, hereafter referred to as the reach limb. Tissues used in this study were from the reach limbs.

### Quantitative real-time PCR (qPCR)

Twelve animals were euthanized with an overdose of sodium pentobarbital (120 mg/kg body weight) and forearm flexor muscles were collected the flexor digitorum mass from FRC, and 3- and 6-week HRNF rats (*n* = 3/group). Each muscle belly was divided into two longitudinal parts; half was used for RNA extraction and half for protein extraction. The half for RNA extraction was put into RNAlater RNA Stabilization Reagent (QIAGEN, Valencia, CA) for 2 h at room temperature and then stored at -80 °C. Total RNA was isolated using TRIzol reagent (Invitrogen, Carlsbad, CA). The concentration of each RNA sample was determined using a spectrophotometer and the integrity was monitored on 1 % formaldehyde denatured gels. After confirming RNA integrity on an agarose gel, cDNA was prepared from mRNA extracts from the above tissue samples (n = 3/group) using a High Capacity cDNA Reverse Transcription kit (Applied Biosystems™, Foster City, CA). PCR primer sets for the following were used: OA (Cat# PPR45879B, SABiosciences, Frederick, MD) and GAPDH (Cat# PPR51520A, SABiosciences, Frederick, MD). Quantitative real time PCT (qPCR) was then performed in duplicate for 20 μl reactions each containing 1 μl cDNA reaction mix, 100nM of each primer and 10 μl 2x SYBR® Green PCR Master Mix method (Applied Biosystems™, Foster City, CA), on an ABI 7500 Fast Real-Time PCR system (Applied Biosystems™). PCR cycles consisted of an initial cycle of 50 °C for 2 min and the second cycle of 95 °C for 10 min, followed by a two-step program of 95 °C for 15 s and 60 °C for 1 min for 40 cycles. Using GAPDH as the internal control, relative gene expression among samples was calculated by comparison of C_t_ (threshold cycle) values. A dissociation curve was checked for each qPCR run to confirm specific amplification of target RNA. The PCR primer set (OA) used was purchased from SABiosciences.

### Protein isolation and western blotting

Animals were euthanized with an overdose of sodium pentobarbital (120 mg/kg body weight) before muscle and tendon collection from the flexor digitorum mass from FRC rats (*n* = 8) and from rats that performed the HRNF task for 3 weeks (*n* = 4) or 6 weeks (*n* = 6). As described above, the muscles were divided into half longitudinally. The half for protein analysis was snap frozen and stored at -80 °C until homogenization and preparation for protein analysis using previously described methods [35, 36]. Total protein was determined using BCA-200 protein assays (Bicinchoninic Acid, Pierce, Rockford, IL). Then, 30 μg of protein sample was mixed with 5X Laemmli sample buffer (Bio-Rad, Hercules, CA) mixed with denaturing buffer, 25 % β-mercaptoethanol, heated to 100 °C for 5 min, subjected to sodium dodecyl sulfate-polyacrylamide gel electrophoresis (SDS-PAGE) and transferred at 100 V for 1 h at 4 °C to an nitrocellulose membrane (Bio-Rad) using semi-dry transfer apparatus (Bio-Rad). The membranes were blocked with 5 % non-fat milk in Tris-buffered saline (TBS)-0.1 % Tween-20 (TBST) for 1 h at room temperature and then incubated with the following primary antibodies: anti-rabbit beta Actin (1:1000; Sigma, St Louis, MO), anti-mouse GAPDH (1:500; Santa Cruz Biotechnology, Delaware Avenue, CA), anti-mouse Hsp72 (1:300; Stressgen, Ann Arbor, MI), anti-rabbit MMP-1 (1:500; Abbiotec, San Diego, CA), anti-mouse MMP-2 (1:200; Abcam, Cambridge, MA), anti-rabbit MMP3 (1:200; Novus Biologicals, Littleton, CO), anti-mouse MMP-13 (1:200; Abcam), anti-rabbit OA/GPNMB (1:500; Bioss Inc. Woburn, MA) and custom made anti-chicken OA (1:250), which was produced as previously described in [37], in blocking buffer (same as above) overnight at 4 °C. The blot was then washed with 1X TBS-0.1 % Tween-20 (TBST) and incubated with the following horseradish peroxidase-conjugated secondary antibodies: donkey anti-chicken, anti-mouse or anti-rabbit (1:5000; Jackson Immunoresearch, West Grove, PA), in blocking buffer for 1 h at room temperature. The blot was washed again with TBST, incubated with SuperSignal West Pico Chemiluminescent Substrate (Thermo Scientific, Rockford, IL) and exposed to film. Quantification of the bands was performed using either Image J or myImageAnalysis v2.0 software (Thermo Scientific).

### ELISA

For protein analysis, tissues from above animals that had been prepared for protein analysis were used in ELISAs to determine HSP72 and inflammatory cytokine levels. All muscle and tendon samples were homogenized separately with 0.5 to 1.0 ml RIPA buffer ((NaCl, KCl, NaH_2_PO_4_, KH_2_PO_4_, and DDH_2_O + NaOH) plus EDTA free complete protease inhibitor cocktail tablets (Roche Diagnostics, GMPH, Germany) using a PowerGen 125 Homogenizer. Tissue homogenates were centrifuged at 14,000 rpm for 15 min. at 4 °C. Total protein was determined using BCA-200 protein assays (Bicinchoninic Acid). For ELISA, tissue lysates (50 microliter aliquots) were analyzed using a commercially available single-plex ELISA kit for HSP72 (EKS-700, Stressgen, purchased before its purchase by Enzo Life Sciences, Inc, Farmingdale, NY), according to the manufacturers’ protocol. Tissue lysates were also analyzed for IL-1alpha, IL-1beta, TNF-alpha and IL-10 using commercially available ELISA kits according to manufacturer’s protocols (each from BioSource International). Each sample was run in duplicate. Data (ng protein of HSP72 and pg protein for the cytokines) were normalized to μg total protein.

### Immunohistochemical analyses and quantification

Animals received an overdose of sodium pentobarbital (120 mg/kg body weight) before being perfused transcardially with first sterile saline and then 4 % paraformaldehyde in 0.1 M phosphate buffer (pH 7.4): FRC (*n* = 6), 3-week HRNF (*n* = 6) and 6-week HRNF (*n* = 4). Tissues were collected and postfixed “en bloc” by immersion overnight. A proximal portion of the muscle mass was then removed with a scalpel for cross-sectional sectioning while the remaining flexor digitorum muscles and tendons were separated as a flexor mass from the bones for longitudinal sectioning, as previously depicted [38]. All tissues were cryoprotected in 30 % sucrose in phosphate buffered saline (PBS) before frozen-sectioning using a cryostat into 15 μm cross sectional or longitudinal slices. Sections were then placed onto charged slides (Fisher, Super Frost Plus) and allowed to dry overnight before storage at -80 °C.

Sections on slides were treated with 3 % H_2_O_2_ in methanol to block for endogenous peroxidase for 30 min (if the tissues were to be used for HRP-DAP visualization), washed in PBS, then permeabilized with 0.05 % pepsin in 0.01 N HCL prior to blocking with 10 % goat serum in PBS for 20 min at room temperature. Sections on slides were then probed in batches with the following primary antibodies: anti-HSP72 (1:1000, C92F3A-5, Enzo Life Sciences, Inc) or anti-osteoactivin (1:350, custom made anti-chicken OA) produced and verified, as previously described [37]. On the 2^nd^ day after washing, one set of sections from all groups that had been incubated with anti-OA antibody was incubated with goat anti-chicken secondary IgG antibody conjugated to HRP (Jackson Immunoresearch Laboratories, West Grove, PA; diluted 1:100 in PBS, and incubated 2 h at room temperature before washing in PBS) and visualized using DAB (Fast DAB, Sigma). These sections were counterstained lightly with eosin, then dehydrated and coverslipped with DPX mounting medium for bright field microscopy (for HRP-DAB). A second set of sections was incubated with anti-HSP72 antibody and then with a donkey anti-mouse secondary IgG antibody conjugated to Cy3 (red fluorescent tag) diluted as above (Jackson Immunoresearch Laboratories). A third set of slides was incubated with both anti-OA antibody and anti-HSP72 and then with appropriate secondary antibodies conjugated to Cy2 (green fluorescent tag) or Cy3 (red fluorescent tag) (Jackson Immunoresearch Laboratories). The fluorescent tag-labeled sections were washed with PBS and coverslipped with 80 % glycerol in PBS for epifluorescence microscopy.

The specificity of the OA and HSP72 antibodies are shown in western blots that are part of this study. In addition, negative control staining was performed by omitting either the primary antibody or the secondary antibody. No labeling was observed as a result of incubation of tissues with serum and then secondary antibodies alone (data not shown). Preabsorption controls were performed to demonstrate if the antibodies bound specifically to the antigen of interest. Specifically, specificity of the OA and HSP72 antibodies was determined via the use of recombinant human Osteoactivin/GPNMB/Fc Chimera (Cat# 2550-AC, R&D Systems, Minneapolis, MN) and recombinant human HSP70 protein (Product# SPP-755, StressGen, Farmingdale, NY; purchased from StressGen before its purchase by Enzo Life Sciences, Inc.), respectively. A ten-fold excess of purified protein was pre-incubated with the matching antibody overnight at 4 °C, the mixture centrifuged and then the pre-absorbed antibody supernatant was incubated with the tissues (after pepsin and goat serum treatments) similar to that described above before washing and incubation with secondary antibodies. No labeling was observed in the tissues for either pre-absorbed antibody (data not shown), matching previously published specificity of the custom made OA antibody in bone tissues [37].

The percent area with OA and HSP72 immunostaining in muscles and tendons was quantified from HRP-DAB stained and fluorescent stained slides, respectively. This quantification was performed using an image analysis program (Bioquant Osteo II, Nashville, TN) and previously described thresholded pixel count methods [39]. The person performing the quantification was blinded to group assignment.

### Statistical analysis

Statistical analyses were performed using Prism 4 and 5 (GraphPad Software, La Jolla, CA). Univariate ANOVAs were used to analyze quantitative PCR, Western blot densitometry and ELISA results for differences in analytes between groups. Then, post hoc analyses were carried out using the Bonferroni test for multiple comparisons with FRC used as the control group and adjusted p values are reported. An adjusted p value of < 0.05 was considered significant for all analyses. Data are presented as the mean ± standard error of the mean (SEM).

## Results

### Increased osteoactivin in flexor digitorum muscles with HRNF task

**Fig. 1:**
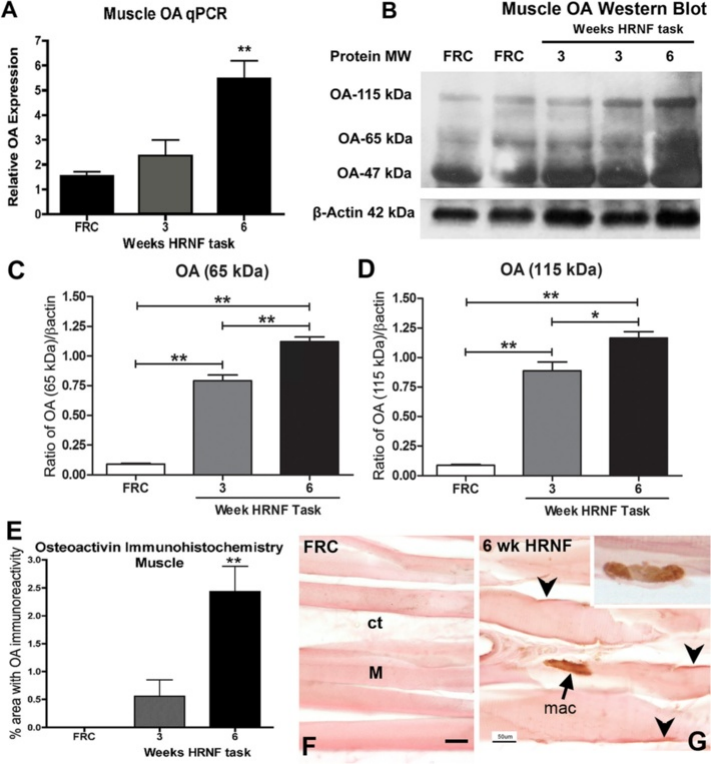
Osteoactivin (OA) expression increases in flexor digitorum muscles with high repetition negligible force (HRNF) task. **a** OA mRNA expression levels (determined using quantitative PCR, qPCR) in muscles of food restricted control (FRC) rats and rats that had performed a HRNF food retrieval task for 3 or 6 weeks. Values were normalized to GAPDH mRNA levels. **b** A representative Western blot of muscle homogenates probed with anti-OA. Bands are at the expected molecular weights of OA in muscle (approximately 115, 65 and 47 kDa). β-actin was used as the loading control (at 42 kDa). **c**, **d** Densitometric analysis of two OA bands (65 and 115 kDa) from three replicate Western blots normalized to β-actin levels. **e** Quantification of OA-positive immunostaining as a percent of total tissue area. **f**, **g** Examples of OA immunostaining in longitudinal sections of FRC and HRNF rat muscles showing OA in the myofiber sarcolemma (arrowheads) and macrophages (mac) only in HRNF muscles. Ct = connective tissue, M = muscle. *p < 0.05, **p < 0.01, compared to FRC rats. Scale bars in F and G = 50 μm

Utilizing quantitative real time PCR (qPCR) analysis, we observed a significant up-regulation of OA gene expression in muscles of 6-week HRNF animals (*p* < 0.01) compared to food restricted only control (FRC) rats (Fig. 1a). These findings were confirmed using Western blot analysis, which showed progressive increases in OA protein levels with HRNF task performance compared to FRC rats (Fig. 1b). The 65 kDa and 115 kDa molecular weight bands of OA were increased (*p* < 0.01) while the 47 kDa band was not increased with HRNF task performance (Fig. 1b-d). To further examine the expression of OA in the muscle tissue of our rat model, we performed immunohistochemistry and subsequent quantification of OA in the flexor digitorum muscles of HRNF rats. OA immunostaining was increased in 6-week HRNF muscles (*p* < 0.01) compared to controls (Fig. 1e). OA was localized to the sarcolemma and to macrophage-like cells located between individual muscle fibers in 6-week HRNF muscles yet was absent in control muscles (Fig. 1f and g).

### MMP-1, -2, -3 and -13 are altered in flexor digitorum muscles with HRNF task

**Fig. 2:**
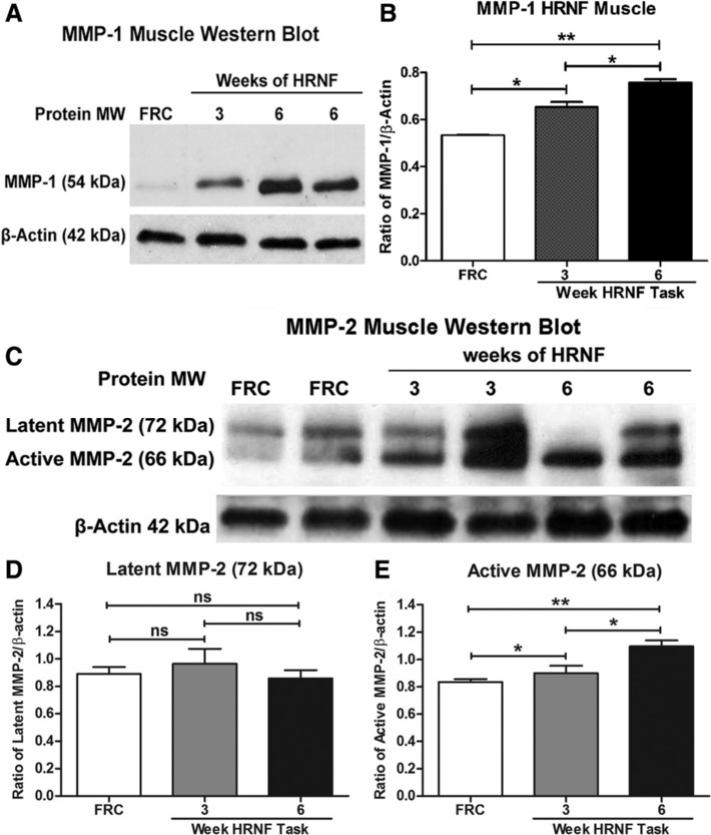
Matrix metalloproteinase (MMP)-1 and -2 protein levels increase in flexor digitorum muscles with high repetition negligible force (HRNF) task. **a** Representative Western blot of muscle homogenates probed with anti-MMP-1. An immunoreactive band at the expected molecular weight of 54 kDa was detected. β-actin was used as a loading control (42 kDa). **b** Densitometric analysis of the MMP-1 band from three replicate Western blots were normalized to β-actin levels and documents significant increases relative to food restricted control (FRC) rats after 3 and 6 weeks of the HRNF task and between 3 and 6 weeks of the task. **c** Representative Western blot of muscle homogenates probed with anti-MMP-2. Bands are detected at the expected molecular weights for the latent (72 kDa) and active (66 kDa) forms. **d**, **e** Densitometric analyses of the MMP-2 protein bands (72 and 66 kDa) from three replicate Western blots after normalization to β-actin levels show that only the active form is increased. *p < 0.05, **p < 0.01, compared to FRC rats; ns = not significant

**Fig. 3:**
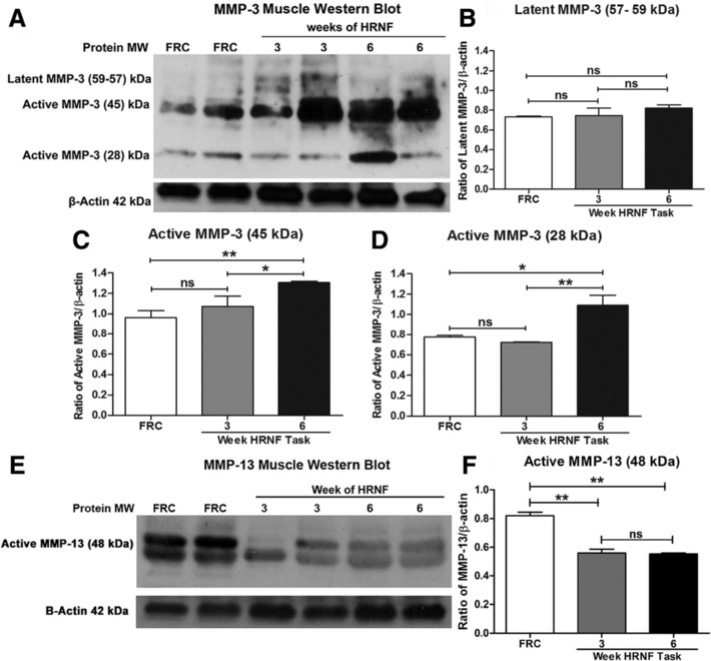
Matrix metalloproteinase (MMP)-3 levels increase whereas MMP-13 decreases in flexor digitorum muscles with high repetition negligible force (HRNF) task. **a** Representative Western blot of muscle homogenates probed with MMP-3. Bands are at the expected molecular weights of latent and active forms of MMP-3 (approximately 57–59, 45, and 28 kDa). β-actin was used as a loading control. **b**-**d** Densitometric analysis of latent (57–59 kDa) and active (45 and 28 kDa) bands of MMP-3 from three replicate Western blots were normalized to β-actin levels. **e** A representative Western blot of muscle homogenates probed with MMP-13. Doublet bands observed at the expected molecular weight of active MMP-13 (approximately 48 kDa) were analyzed, averaged and normalized to the loading control. **f** Densitometric analysis of active (48 kDa) form of MMP-13 from three replicate Western blots after normalization to β-actin levels shows that it decreased significantly with the HRNF task. *p < 0.05, **p < 0.01, compared to FRC rats; ns = not significant

Western blotting detection showed that MMP-1, -2 and -3 were significantly increased in 3- and 6-week HRNF rat muscles compared to FRC rats (Figs. 2 and 3). Specifically, the 54 kDa molecular weight band of MMP-1 (Fig. 2a and b) and the 66 kDa active form of MMP-2 (Fig. 2c and e) were increased in HRNF rat muscles (*p* < 0.05 each), particularly by 6 weeks of task performance. The 72 kDa latent form of MMP-2 did not change significantly across groups (Fig. 2c and d). Expression levels of the active forms of MMP-3 (45 kDa and 28 kDa) also increased significantly in 6-week HRNF rats (*p* < 0.05 each) compared to 3-week HRNF and FRC rat muscles (Fig. 3a, c and d). In contrast, the latent form of MMP-3 (57–59 kDa) did not change (Fig. 3a and c), and the level of active MMP-13 (48 kDa) decreased with HRNF task performance (*p* < 0.01) compared to controls (Fig. 3e and f).

### HSP72 increases in muscles and Co-localizes with OA in sarcolemma of HRNF rats

**Fig. 4:**
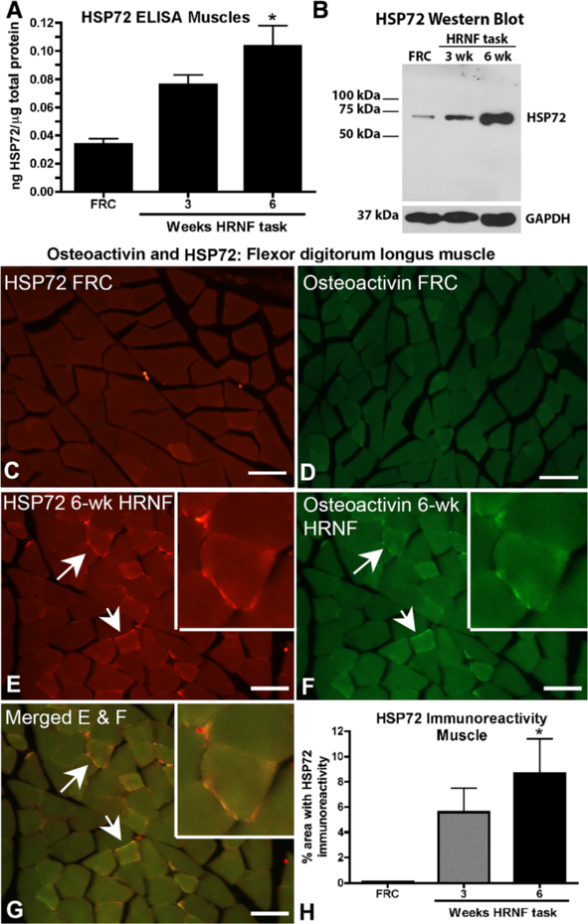
Heat shock protein 72 (HSP72) increases in flexor digitorum muscles of high repetition negligible force (HRNF) rats and co-localizes with osteoactivin (OA) in the sarcolemma. **a** ELISA of HSP72 protein levels in FRC and HRNF rat muscle lysates. Nanograms of protein were normalized to μg of total protein. **b** Representative Western blots of muscle homogenates probed with anti-HSP72 were used to confirm that the molecular weight of the HSP protein recognized by the antibody was the inducible form of HSP72. **c** Immunostained image of HSP72 (red) in muscle from a food restricted control (FRC) rat (a proximal portion of the muscle was resected and cut transaxially and shown), showing that there was little to no immunostaining. **d** Immunostained images of OA (green) in muscle from a FRC rat (a proximal portion of the muscle was resected and cut transaxially and shown) showing little to no immunostaining. **e**-**g** Double-labeled immunostained images of HSP72 (red) and OA (green) were merged in muscle images from an 8-week HRNF rat (a proximal portion of the muscle was cut transaxially and is shown). Arrows indicate HSP72 and OA co-localization in myofiber sarcolemma. The insets in e-g show enlarged regions of these images to illustrate co-localization of HSP72 and OA in myofiber sarcolemma. **h** Quantification of HSP72-positive immunostaining as a percent of total tissue area. *p < 0.05, compared to FRC rats. Scale bars in C-G = 50 μm

ELISA analysis showed significant increased HSP72 protein in 6-week HRNF muscles (*p* < 0.05) compared to controls (Fig. 4a). This result was supported by Western blot analysis that confirmed the expression and increase of the HSP72 protein in 6-wk HRNF rat muscles compared to controls (Fig 4b). We also evaluated the expression of OA and HSP72 using immunohistochemical methods. Little to no HSP72 or osteoactivin was observed in FRC rat muscles (representative examples shown in Fig. 4c and d). Double labeling immunohistochemistry in 6-week flexor digitorum muscles demonstrated increased expression of HSP72 (in red; Fig. 4e, g) and OA (in green; Fig. 4f, g), with co-localization in the sarcolemma surrounding the myofibers (Fig. 4g). Quantification of HSP72 immunostaining confirmed its increased immunoexpression in 6 week HRNF rat muscles compared to controls (Fig. 4h). No DAPI labeled cell bodies were visualized within the center of any of the myofibers at this 6-week HRNF time point (data not shown), indicating that the myofibers were not injured.

### Osteoactivin and HSP72 increase in tendons with HRNF task

**Fig. 5:**
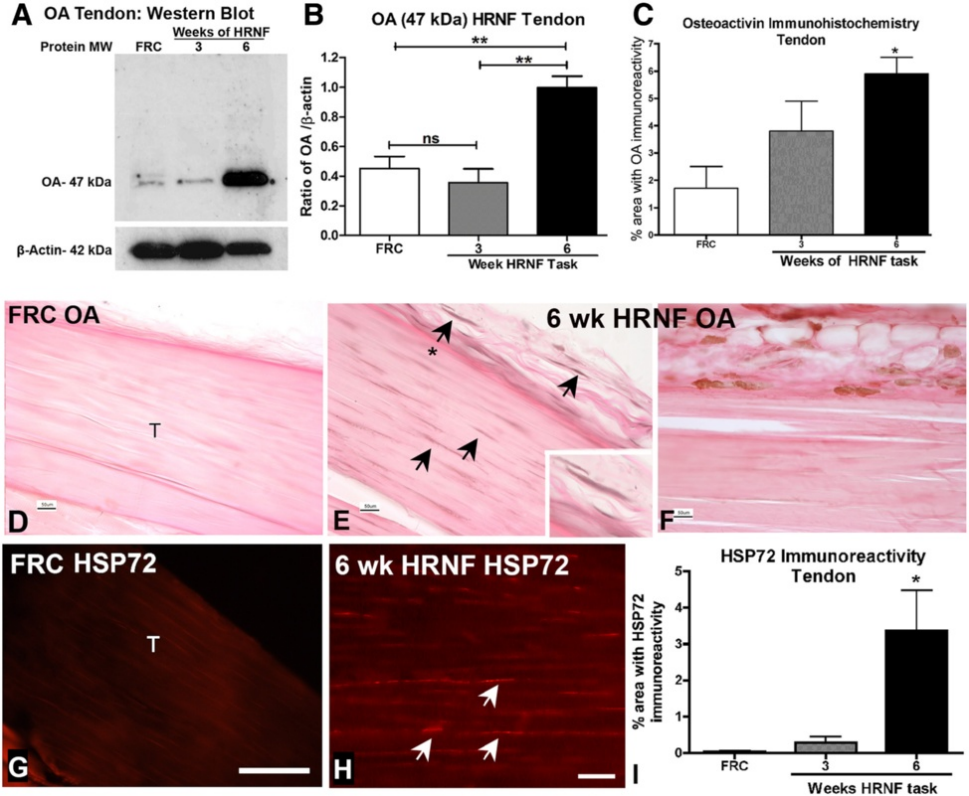
Flexor digitorum tendons show increased osteoactivin (OA) and heat shock protein 72 (HSP72) by six weeks of high repetition negligible force (HRNF) task performance. **a** Representative Western blot of tendon homogenates probed with anti-OA. Band detected is the 47 kDa MW. β-actin was used as a loading control (at 42 kDa). **b** Densitometric analysis of the 47 kDA OA band in tendon from three replicate Western blots after normalization to β-actin levels. **c**-**f** Immunostaining and quantification of OA in food restricted control (FRC) and HRNF rat tendons (T). A tendon region located proximal to the wrist and cryosectioned longitudinally, is shown. Arrows indicate representative OA-immunopositive cells, many of which are tenocyte-like in appearance. Fibroblast-like cells were also observed in the epitendon (one indicated with an asterisk in panel E and enlarged in the inset) as well as macrophage-like cells (arrow heads). **g**-**i** Immunostaining and quantification of HSP72 in FRC and HRNF rat tendons (T). Arrows indicate representative HSP72-immunopositive cells that appear tenocyte-like. *p < 0.05, **p < 0.01, compared to FRC rats. Scale bars in D-F,H = 50 μm; Scale bar in G = 100 μm. n.s. = not significant.

Next, we examined the expression of OA and HSP72 in flexor digitorum tendons. Western blot analysis showed that only the 47 kDa form of OA was increased in tendons of 6-week HRNF task performance (*p* < 0.01) compared to FRC rats (Fig. 5a and b). Immunoexpression of OA and its quantification showed significantly increased OA immunostaining in 6-week HRNF tendons (*p* < 0.05) compared to control rat tendons (Fig. 5c-f). OA was localized to tenocytes within 6-week HRNF tendons and to fibroblast- and macrophage-like cells located within the surrounding connective tissue (epitendon), but was absent in control tendons and epitendon (Fig. 5d-f ). Similarly, HSP72 immunostaining was significantly increased in tenocytes located within 6-week HRNF tendons compared to controls (Fig. 5g-i).

### MMP-1 and -3 increase in tendons with HRNF task

**Fig. 6:**
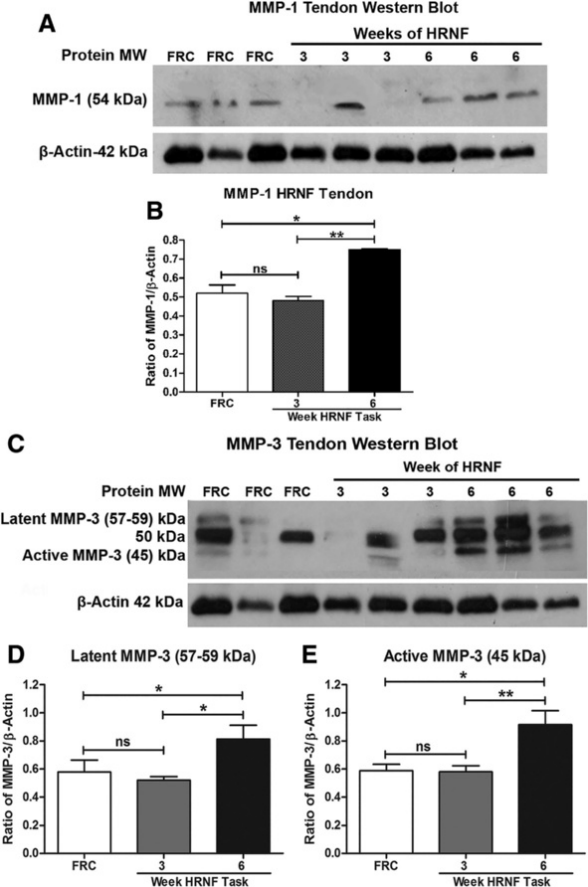
Matrix metalloproteinase (MMP)-1 and -3 protein levels increase in flexor digitorum tendons with high repetition negligible force (HRNF) task. **a** Representative Western blot of tendon homogenates probed with anti-MMP-1. Bands are at the expected molecular weight of 54 kDa. β-actin used as a loading control (at 42 kDa). **b** Densitometric analysis of three replicate Western blots shows the ratio of the MMP1 band after normalization to β-actin levels. **c** A representative Western blot of muscle homogenates probed with MMP-3. Bands are at the expected molecular weights of latent and active forms of MMP-3 (approximately 57–59 and 45). β-actin used as a loading control. **d**, **e** Densitometric analysis of latent (57–59 kDa) and active (45 kDa) forms of MMP-3 from three replicate Western blots after normalization to β-actin levels. *p < 0.05, **p < 0.01, compared to FRC and 3 week rats. n.s. = not significant.

Western blot analysis showed the level of MMP-1 (54 kDa) was increased in 6-week HRNF rat tendons (*p* < 0.05), compared to 3-week HRNF and FRC rat tendons (Fig. 6a and b). Note the variability of MMP-1 in the 3-week HRNF tendons (Fig. 6a). This was a consistent finding. We next found that the latent (57–59 kDa) and active (45 kDa) forms of MMP-3 were increased significantly in 6-week HRNF rat tendons (*p* < 0.05) compared to 3-week HRNF and FRC rats (Fig. 6c-e). Variable expression of the latent forms of MMP-3 was observed in FRC and 3-week HRNF tendons, while the active form of MMP-3 (45 kDa) was consistently increased in the 6-week HRNF tendons.

### IL-1alpha and IL-1beta alter across time in muscles and tendons with HRNF Task

**Fig. 7:**
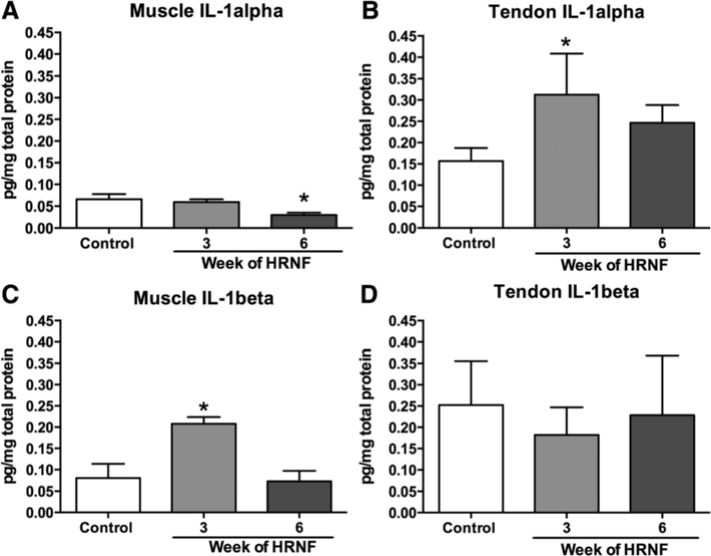
ELISA of inflammatory cytokines in flexor digitorum muscles and tendons of reach limb. Data is shown for (**a**, **b**) interleukin (IL-1) alpha and (**c**, **d**) IL-1beta in food restricted control (FRC) and 3- and 6-week HRNF rats. *p < 0.05, compared to FRC rats

Lastly, we also observed transient yet significant increases of IL-1alpha in 3-week HRNF tendons (Fig. 7b) and IL-1beta in 3-week HRNF muscles (Fig. 7c). We also observed a very small but significant decrease in IL-1alpha in 6-week HRNF muscles compared to controls (Fig 7a). No increase was observed for IL-1beta in tendons (Fig. 7d). TNF-alpha and IL-10 did not alter significantly with task performance in either tissue compared to controls (data not shown).

## Discussion

For the first time to our knowledge, we show in a rat model of limb overuse that expression of the growth factor osteoactivin (OA) increases progressively in forearm muscles and tendons with prolonged performance of an upper extremity high repetition negligible force (HRNF) task for up to 6 weeks. Immunostaining also showed localization of OA to myofiber sarcolemma, macrophage-like cells, fibroblast-like cells and tenocytes. Levels of enzymatic proteins related to tissue turnover, MMP-1, -2 and -3, also progressively increased, whereas MMP-13 decreased, in forearm muscles with HRNF task performance. MMP-1 and -3 levels also progressively increased in forearm tendons. Levels of a known repair protein, HSP72, also increased only in 6-week HRNF muscles and tendons compared to controls, and co-localized with OA immunoexpression in the sarcolemma of 6-week HRNF muscles.

We have previously shown modest muscle and tendon injury, followed by a low-grade but transient inflammatory response, with performance of this HRNF reaching and food retrieval task [9, 35]. Since inflammation occurring in injured tissues as a consequence of overuse is thought to be related to repair processes [40], we extended our past studies to examine repair and remodeling factors in this current study. Repair of musculoskeletal tissues may be driven by a number of anabolic factors. We examined OA because it has been identified as an anabolic growth factor in long bones [41], and found that it was increased in muscles and tendons of HRNF rats, with the greatest increases in parallel with a return of inflammatory cytokines to baseline levels. The increase in OA was concomitant with increased MMP and HSP72, proteins known to participate in repair and remodeling processes, thus further supporting a role for OA as an anabolic molecule. Several studies have shown that OA regulates cell proliferation, adhesion, differentiation and synthesis of extracellular matrix proteins in various cell types under normal and repair/regeneration conditions [16, 41–49]. In skeletal muscle, OA increases after denervation or distraction injury [14–16, 50] and has been shown to up-regulate MMP-3 and -9 expression in fibroblasts infiltrating denervated skeletal muscle [16]. Extracellular fragments of OA produced by ectodomain shedding induce MMP-3 expression in myofibroblasts after unloading [14]. These latter responses serve to enhance tissue turnover and repair.

Tissue injury is known to induce the expression of matrix-related genes, including MMPs [51–54]. Therefore, we examined their expression and observed that performance of the HRNF task for 6 weeks increased MMP expression and found that MMP-1 (collagenase), -2 (gelatinase) and -3 (stromelysin-1) were increased in flexor digitorum muscles, while MMP-1 and -3 were increased in tendons. We have previously shown that MMP-2 increases in serum, forearm muscles and tendons of rats performing a high repetition low force lever-pulling task for 18 weeks [55]. MMPs are zinc-dependent proteases that regulate cell-matrix composition, modulate ECM turnover, and are produced by fibroblasts, mesenchymal cells and macrophages [56–59]. The expression of most MMPs is low in normal steady-state tissues and are induced only when ECM remodeling is needed [60], such as for musculoskeletal tissue adaptability to loading and training [61–63]. Their expression is transcriptionally controlled by growth factors, cell-cell and cell-matrix interactions [64–66] and inflammatory cytokines [62, 63]. Examination of an experimental model of muscle regeneration [cardiotoxin (CTX) injection] shows a tightly regulated time course of MMP activation and resolution of tissue damage [52, 67, 68]. Also, generalized MMP inhibition impairs muscle repair [69] further supporting their requirement in resolution of muscle damage [70].

In tendons, MMPs are involved in collagen catabolism and show increased activity with prolonged periods of high or low mechanical loading or during periods of tendon repair [71–73]. Studies have shown increased MMP-1 or MMP-2 with chronic loading of tendons [74] in tendons with signs of overuse injury [75, 76] and in flexor tendosynovial tissues collected from patients with carpal tunnel syndrome [77]. In agreement with our data, a synergistic effect of mechanical stretch and presence of inflammatory cytokines has been shown to induce MMP-1 and -3 expression in tendon cells more than stretch alone [78–80]. The increased MMPs with HRNF task performance are likely responding to the continued loading and transient increases of IL-1alpha in tendons and IL-1beta in muscles and are contributing to tissue remodeling for repair.

Since previous studies suggest that heat shock proteins (HSPs) play a role in skeletal muscle repair after high-force eccentric exercise and that elevated HSP70 and its inducible form (HSP72) protects skeletal muscle against further injury [28, 29], we examined its expression and found that this stress-inducible protein was increased in 6-week HRNF muscles compared to controls. HSP72 plays a role in skeletal muscle remodeling and adaptation processes in response to exercise and stress [81, 82]. Exercise training increases the expression of HSP72 a few days after the onset of training [83–85] while prolonged exercise training, using intermittent high-intensity treadmill running for 8 weeks, induces long-term enhancement of HSP70 expression in skeletal muscle [86]. Interestingly, Sjogaard et al. showed in human subjects that performance of repetitive tasks increased HSP72 in muscles while prolonged exercise training decreased its basal levels [87], suggestive of a clear difference between these two activities. Activation of HSP72 may play a dual role in inflammation [88], inhibiting the release of inflammatory cytokines including IL-1beta [89–93]. This interaction between HSP72 and inflammatory cytokines may explain our findings of only low levels of IL-1alpha and beta by 6 weeks of task performance.

As mentioned above, since HSP72 stimulate anti-inflammatory cytokines and inhibit the release of some inflammatory cytokines, we next examined the muscles and tendons for presence of inflammatory cytokines. We observed that performance of a HRNF task for 3 weeks induces significant increases in IL-1alpha in tendons and IL-1beta in muscles, yet declines to baseline or even below baseline levels by 6 weeks of task performance. Since pro-inflammatory cytokine expression are induced at early time points during tissue repair [94], we suggest that the 3 week time point is the peak of inflammatory phase in this high repetition negligible force reaching and grasping model, and that the 6-week time point is the beginning of the repair proliferative phase, with HSP72 and OA mediating tissue repair and adaptation.

## Conclusions

These findings suggest that performance of a high repetition negligible force reaching and grasping induces an inflammatory response at 3 weeks and then a repair response at 6 weeks that might be mediated, at least in part, by OA, MMPs and HSP72. Further research is needed to determine the production of these proteins in musculotendinous tissues undergoing higher levels of persistent overloading and tissue injury, such as in animals and humans performing a high repetition high force task for longer periods of time.
